# Prescription drug coverage and effective coverage of three chronic conditions of high prevalence in Chile: Hypertension, diabetes and dyslipidemia

**DOI:** 10.1371/journal.pone.0297807

**Published:** 2024-02-12

**Authors:** Isabel Matute, Carla Castillo-Laborde

**Affiliations:** Centro de Epidemiología y Políticas de Salud, Facultad de Medicina Clínica Alemana, Universidad del Desarrollo, Santiago, Chile; Universidad de Caldas, COLOMBIA

## Abstract

**Background:**

Access to medicines is a serious problem globally and in Chile. Despite the creation of coverage policies, part of the population with chronic conditions of high prevalence, still does not have access to the medicines it requires and disease control continues to be low. The objective of the study was to estimate the medication use and effective coverage for diabetes, dyslipidemia and hypertension in Chile, analyzing them according to sociodemographic variables and social determinants of health.

**Methods:**

Cross-sectional analytical study with information from the 2016–2017 National Health Survey (sample = 6,233 people aged 15 years or older, expanded = 14,518,969). Descriptive analyses of medication use and effective coverage for hypertension, diabetes and dyslipidemia were carried out, and multivariate logistic regression models were developed to analyze possible associations with variables of interest.

**Results:**

60% of people with hypertension or diabetes use medications and only 27.7% in dyslipidemia. While 54.2% of those with diabetes have their glycemia controlled, in hypertension and dyslipidemia the effective coverage drops to 33.3% and 6.6%, respectively. There are no differences in use by health system, but there are differences in the control of hypertension and diabetes, favoring beneficiaries of the private subsystem. Effective coverage of dyslipidemia and hypertension also increases in those using medications. The drugs coincide with the established protocols, although beneficiaries of the private sector report greater use of innovative drugs.

**Conclusion:**

A significant proportion of Chileans with hypertension, diabetes or dyslipidemia still do not use the required medications and do not control their conditions.

## Introduction

Three of the most prevalent chronic conditions in the world are diabetes, dyslipidemia and hypertension, whose treatment and control are associated with medication use [1–3]. However, lack of access to essential medicines, such as those required for these diseases, remains one of the most important public health problems globally, with an estimated third of the population lacking regular access to them [4].

The barriers that hinder this access include their availability, geographical distances to care centers, the cost of medicines, the lack of support by health professionals, and cultural aspects linked to their acceptability [5], which is exacerbated by the particularities of the drug market, such as the existence of monopolistic powers, brand loyalty, or the structure of demand at multiple levels, including its assurance [6].

These difficulties are certainly relevant and determine the medication use [7] given, in this case, by the proportion of people in the target population who use medications for their condition. However, use does not ensure the success of the intervention, especially in the case of chronic diseases such as those studied, which require continuity and adherence. In this context, Tanahashi introduces the concept of effective coverage, understood as the proportion of the target population that receives a satisfactory or effective service, which is reflected in the control of their medical condition [7]. The difference between use and effective coverage is evident in cascade analyses, thus, for example, a study conducted in the United States recorded that 10.7 million adults did not know they had hypertension; 3.8 million knew this but did not treat their disease; and 15.8 million treated it but did not control it [8].

In addition, knowledge, treatment and control outcomes vary substantially across countries [9] and between urban and rural settings [10].

In the case of Chile, about 58% of the population over 15 years of age consumes at least one medication, the most frequent being paracetamol and acetylsalicylic acid, followed by losartan, metformin and atorvastatin [11]. On the other hand, among those who presented a health problem and received medical attention, 7.4% reported having had difficulties accessing medicines [12], either because they were not available at health facilities or it was not possible to buy them due to their cost, reflecting the relevance of out-of-pocket spending as a barrier to access in the country. In fact, in Chile, medicines are the most important item of out-of-pocket health spending [13], and at least 30% of people have stopped buying medicines or suspended their treatments because they cannot afford them [14].

During the last decades, two important strategies have been developed to increase coverage for the three conditions analyzed, the Explicit Health Guarantees (GES, for its acronym in Spanish), in the case of diabetes and hypertension [15] and the Pharmacy Fund for Chronic Noncommunicable Diseases in Primary Health Care (FOFAR, for its acronym in Spanish), which provides financing for these two diseases and for dyslipidemia, but only in the public health system [16].

However, the prevalence of these diseases remains high, with values of 46% in dyslipidemia, 28% in hypertension and 12% in diabetes [17]. And estimates of effective coverage for hypertension (17%) and diabetes (35%) in 2009 are very low [18], as well as in 2016 (33% and 58%, respectively) [19, 20]. This leaves ample room for improvement and translates into an important challenge for the health system. Even more so considering the complexity involved in the segmentation of the Chilean system, which means that the mechanisms for access to medicines differ depending on whether it is the public (FONASA) or private (ISAPRE) health subsystem [21, 22].

Despite the relevance of this issue, there is limited evidence on recent trends in the cascade of care for these diseases in countries like Chile [10]. Therefore, the objective of this study was to estimate the medication use and effective coverage for diabetes, dyslipidemia, and hypertension in Chile, analyzing them based on sociodemographic variables and social determinants of health, as well as characterizing medication usage.

## Materials and methods

### Study type and data source

A cross-sectional analytical study was conducted based on information provided by the National Health Survey (NHS), in its 2016–2017 version. This population survey, commissioned by the Ministry of Health and executed by the Pontificia Universidad Católica de Chile, corresponds to a cross-sectional design.

### Population and sample

The universe considered the population aged 15 years or older residing in Chile, with a sample of 6,233 respondents (expanded = 14,518,969), of which 5,520 had laboratory tests and 5,514 answered the "Medication Use" module [23]. The study used a complex, multistage (strata and clusters) and random sampling at all stages, with national, regional, urban/rural representativeness [23]. This sample allowed the calculation of prevalence of diabetes, dyslipidemia and hypertension, among other health conditions. For specific calculations on medication use and effective coverage, we worked with subsamples that presented the diseases studied (e.g. for diabetes, the subsample includes only those people who met the diabetes criteria definition).

### Variables and instruments

The 2016–2017 version incorporated questions, anthropometric, biophysiological and laboratory tests associated with 84 health problems, social determinants, risk factors and protective factors [24]. In addition, it included a "Medication Use" module with detailed information on the medications used by the respondents at the time of the survey.

The prevalences were defined based on the criteria established by the NHS 2016–2017 [25]:

- Hypertension (AHT): people who self-reported having AHT or medical treatment for AHT or with elevated pressure greater than 140/90 (average with 3 blood pressure readings).
- Diabetes (DM): people who self-reported having DM or elevated glycemia greater than or equal to 126 mg/dl fasting, excluding gestational diabetes.
- Dyslipidemia (DLP): in this case, the NHS criteria were adapted to match Chilean clinical guidelines in terms of pharmacological treatment need [26]. People with high LDL cholesterol (greater than 100 mg/dL) or people with self-reported medications, and with high cardiovascular risk or self-reported previous myocardial infarction were included.

Medication use was measured as the proportion of people in the target population (meet the prevalence criteria definition) who is under pharmacological treatment for it (using medicines). Effective coverage represents the proportion of the target population receiving a satisfactory or effective service, which is reflected in the control of their health problem [7]:

- AHT: pressure lower than 140/90 (average with 3 blood pressure readings).
- DM: glycemia lower than 126 mg/dl fasting.
- DLP: LDL cholesterol lower than 70 mg/dL (defined according to the clinical guidelines for individuals with high cardiovascular risk).

The analysis also considered demographic variables: sex (male/female), age group (15–24, 25–44, 45–64 or 65 and older), and residential area (rural/urban); and variables associated with social determinants of health: having or not having a partner, belonging or not to an indigenous people, education level (<8 years, 8–12 years or >12 years of study), and health system (public/FONASA, private/ISAPRE or other situation).

Regarding medicines, the survey allowed to describe their current use, considering their Anatomical Therapeutic Chemical (ATC) Classification, their commercial name, who prescribed it (general practitioner, psychiatrist, other specialist physician, dentist, midwife, pharmacist, him/herself or another person) and the method of acquisition (in the clinic/hospital/public system network, pharmacy with ISAPRE card or clinic, retail pharmacy, another place, was given to him by an acquaintance, through GES or other) [25].

### Analysis

First, a descriptive analysis (relative frequencies and confidence intervals) of prevalences, medication use and effective coverage for the three health conditions was conducted. Second, in order to analyze possible associations between medication use (dependent variable 1: use; 0: no use) and effective coverage (dependent variable 1: control; 0 no control) with other variables of interest, multivariate logistic regression models were developed, considering a p value <0.05. Third, medication usage was characterized, identifying the most used medications for each of the three health conditions, and analyzing those medications regarding their prescription (by whom) and acquisition (how).

Finally, the coincidence of medication use with the protocols established for each disease was reviewed.

The analysis was conducted by the authors with the SPSS statistical software using the expanded sample.

### Ethical considerations

Only data from secondary sources were used in this study. However, it is part of the FONIS Project SA19|0174, which was approved by the Bioethics Committee of the Faculty of Medicine of Clínica Alemana Universidad del Desarrollo.

## Results

### Prevalence of the conditions studied

Based on the measurements taken in the last National Health Survey (NHS 2016–2017), the prevalence was calculated for each condition studied for the population aged 15 years and over residing in Chile, obtaining 12.3% for Diabetes Mellitus (DM), 27.6% for Hypertension (AHT) and 14.1% in the case of Dyslipidemia (Table 1). It should be noted that about 855,267 people had both DM and dyslipidemia (approximately 6%), 1,035,764 had DM and AHT (7%), 1,117,813 had dyslipidemia and AHT (7.7%), and 529,897 (3.6%) had all three conditions.

**Table 1:** Prevalence of Diabetes Mellitus, dyslipidemia and hypertension in people over 14 years of age in Chile, according to NHS measurements 2016–2017.

	Prevalence	Medication use	Effective coverage
	%	%	%
	95%CI	95%CI	95%CI
Diabetes	**12.3**	**60.7**	**54.2**
(N = 13,773,403^a^)	11.1–13.8	55.2–66.0	47.9–60.3
Dyslipidemia	**14.1**	**27.7**	**6.6**
(N = 13,660,519^a^)	12.5–15.9	22.1–34.0	4.1–10.3
Hypertension	**27.6**	**59.9**	**33.3**
(N = 14,501,548^a^)	25.7–29.7	55.9–63.8	29.5–37.2

Source: Prepared by authors, based on NHS database 2016/2017.

^a^Expanded sample.

Those who presented DM (n = 1,700,422) were mainly women (58.1%, n = 987,588), their mean age was 57 years (95%CI 55–58.8 years; range 15–95 years), 80.9% (n = 1,364,737) were beneficiaries of the Public Health System (FONASA), 88.9% (n = 1,511,441) resided in urban areas and 37.8% (n = 642,369) resided in the Metropolitan Region or capital of Chile.

In the case of people with dyslipidemia (n = 1,923,146), 57.8% (95%CI 50.6% - 64.6%) were women, the mean age reached 58.2 years (95%CI 56.02–60.41 years; range 16–98 years), 83.0% (95%CI 76.5% - 87.9%) were affiliated to FONASA, 87.1% lived in urban areas and 39.0% resided in the Metropolitan Region.

On the other hand, when considering those with hypertension (n = 4,004,957), 51% (n = 2,042,812) were women, a mean age of 60 years was recorded (95%CI 58.8–61 years; range 19–98 years), 84.7% (n = 3,363,645) were FONASA beneficiaries, 87.1% (n = 3,489,844) resided in urban areas and 36.6% (n = 1,466,905) lived in the capital of the country.

### Medication use

Regarding the use of medications, this was recorded in 60.7% of those with DM, in 59.9% of people with hypertension and only in 27.7% of those with dyslipidemia (Table 1).

When analyzing, through multivariate models, different factors that may be associated with the use of medications in each condition studied (Table 2), it was observed, in the case of DM, greater use in women, lower use in lower age groups compared to people 65 years or older and in persons belonging to indigenous peoples. In addition, an educational gradient was recorded, with an increase in use with increasing years of study.

**Table 2:** Use of medications and associated factors for Diabetes Mellitus, dyslipidemia and hypertension in people over 14 years of age in Chile, according to NHS measurements 2016–2017.

Variable	Category	DM	DLP	AHT
Sex (ref. male)	Female	1.734**	3.132***	2.512***
		1.076–2.794	1.704–5.758	1.725–3.657
Age group (ref. >65)	15–24 years	0.094***	1.40E-12***	1.034
		0.022–0.402	4.92E-13–3.96E-12	0.206–5.182
	25–44 years	0.231***	0.142***	0.257***
		0.104–0.510	0.039–0.514	0.133–0.497
	45–64 years	0.600*	0.836	0.891
		0.355–1.016	0.463–1.508	0.603–1.316
Ethnicity (ref. Not belong)	Indigenous people (Yes)	0.412**	1.046	0.686
		0.187–0.909	0,431–2.538	0.362–1.299
Marital status (ref. with partner)	No partner	0.896	0.905	0.747
		0.547–1.467	0.478–1.714	0.518–1.078
Health System (ref. FONASA)	Other	0.326*	0.765	1.162
		0.106–1.005	0.150–3.903	0.554–2.438
	ISAPRE	2.135	0.590	1.333
		0.646–7.055	0.228–1.524	0.624–2.850
Educational Level (ref. <8 years)	8–12 years	1.766*	1.350	1.343
		1.002–3.115	0.724–2.518	0.906–1.990
	13 years or more	2.541*	1.649	2.389***
		0.929–6.945	0.599–4.543	1.294–4.410
Area (ref. urban)	Rural	1.203	1.823*	1.288
		0.755–1.917	0.959–3.463	0.842–1.971
n		845	719	1,960
Population size		1,680,145	1,913,382	3,922,193
Prob > F		0.001	0.000	0.000
Nagelkerke		0.141	0.153	0.130

Source: Prepared by authors, based on NHS database 2016/2017.

* p<0.1

** p<0.05

*** p<0.01.

In dyslipidemia, association of medication use with sex and age was recorded; greater use in women, and the group aged 25 to 44 years had lower utilization than people aged 65 years or older.

In AHT, a greater use was observed in women and in people with 13 or more years of education compared to those with less than 8 years of schooling. In terms of age, lower use was observed in the 25 to 44 age group compared to people aged 65 years and older.

### Effective coverage

Effective coverage was higher in the case of DM, where 54.2% had their disease controlled. In AHT this figure fell to 33.3%, and reached only 6.6% in dyslipidemia (Table 1).

In terms of the associated factors (Table 3), DM showed lower compensation in the 45–64 age group compared to people aged 65 years or older and greater effective coverage in ISAPRE members compared to those in FONASA.

**Table 3:** Effective coverage and associated factors for Diabetes Mellitus, dyslipidemia and hypertension in people over 14 years of age in Chile, according to NHS 2016–2017 measurements.

Variable	Category	DM	DLP	AHT
Sex (ref. male)	Female	1.309	1.383	2.549***
		0.790–2.168	0.468–4.088	1.700–3.821
Age group (ref. >65)	15–24 years	-	1.51E-11***	8.343***
			5.00E-12–4.56E-11	1.660–41.943
	25–44 years	1.781	1.31E-11***	1.161
		0.650–4.881	5.60E-12–3.05E-11	0.567–2.374
	45–64 years	0.456***	1.155	2.424***
		0.267–0.780	0.453–2.941	1.675–3.507
Ethnicity (ref. Not belong)	Indigenous people (Yes)	0.749	1.629	0.755
		0.204–2.746	0.485–5.472	0.38–1.501
Marital status (ref. with partner)	No partner	0.941	0.836	0.586***
		0.538–1.646	0.282–2.474	0.411–0.836
Health System (ref. FONASA)	Other	0.916	3.148	0.889
		0.269–3.119	0.490–20.228	0.425–1.859
	ISAPRE	4.247***	0.503	2.162**
		1.542–11.698	0.099–2.57	1.148–4.072
Educational Level (ref. <8 years)	8–12 years	1.370	1.167	1.369
		0.727–2.583	0.463–2.945	0.895–2.093
	13 years or more	1.062	0.503	2.109**
		0.405–2.784	0.126–2.018	1.144–3.889
Area (ref. urban)	Rural	0.760	1.149	1.070
		0.391–1.478	0.413–3.201	0.745–1.537
n		748	719	1,959
Population size		1,456,243	1,904,224	3,921,414
Prob > F		0.000	0.000	0.000
Nagelkerke		0.184	0.098	0.148

Source: Prepared by authors, based on NHS database 2016/2017.

** p<0.05

*** p<0.01

Regarding dyslipidemia, the associations focused on age: the groups of 15–24 years and 25 to 44 years had a lower compensation or control of the disease than people 65 years or older, but with OR close to 0.

In the case of AHT, effective coverage was higher in women, in the groups of 15 to 24 years and 45 to 64 years in contrast with those older than 64 years, in people with 13 or more years of education compared to those with less than 8 years of education and in ISAPRE members relative to those of FONASA. On the other hand, compensation was lower in people without a partner.

Finally, when analyzing the effective coverage by medication use, there is a significant difference in both dyslipidemia and AHT. While effective coverage is 0% in people who do not use them, it increases to 24.0% (95%CI 15.2–35.7%) in those who use medications for dyslipidemia and 55.5% (95%CI 50.6–60.3%) in those who do so for AHT. In DM, although there is a trend in favor of medication use (57.1% effective coverage in people with medications 95%CI 48.3–65.4%; and 50% in people without medications 95%CI 40.8–59.2%), this is not significant.

### Characterization of medication usage

When considering those who presented DM and took medications for their condition, Metformin (A10BA02), belonging to the biguanides and which helps control the glucose levels in the blood [27], was used by 34.6% of the people, with close values among members of FONASA (35.3%) and ISAPRE (38.2%). On the other hand, Glibenclamide (A10BB01), which stimulates insulin secretion [27], was consumed by 5.6%, which amounted to 6.4% when only considering FONASA beneficiaries, since its reported use was very low in ISAPRE members (2.6%). Insulin (human) (A10AC01), a soluble analogue of insulin [27], was used by 2% of those with DM and was only reported by FONASA beneficiaries (2.5%).

In most cases, these medications were prescribed by a general practitioner; however, while this trend is clear in the case of FONASA, in ISAPRE members the prescription came from specialist physicians. Something similar happened regarding the way they were obtained, since most people received them in the public health network, even reaching more than 90% in the FONASA beneficiaries, however, in those who had ISAPRE, acquisition in pharmacies appeared as a relevant alternative. In both FONASA and ISAPRE, there was a low mention of obtaining them through GES (Table 4).

**Table 4:** Prescription and acquisition of medicines for Diabetes Mellitus, dyslipidemia and hypertension in people over 14 years of age in Chile by the health system, according to NHS measurements 2016–2017.

Prescription and obtaining		DM			Dyslipidemia		AHT		
		Metformin	Glibenclamide	Insulin	Atorvastatin	Gemfibrozil	Losartan	Enalapril	Valsartan
All health systems									
Who prescribed this medication?	General practitioner	**76.7**	**93.6**	**76**	**68.7**	**100**	**87.9**	**98.3**	**64.6**
		57.2–89.0	56.1–99.4	52.4–90.1	28.2–92.4	100–100	76.6–94.1	95.4–99.3	35.9–85.6
	Other specialist physician	**23.3**	**6.4**	**24.0**	**8.3**	**-**	**11.8**	**1.7**	**30.9**
		11.0–42.8	0.6–43.9	9.9–47.6	0.6–59.4		5.6–23.2	0.7–4.6	11.2–61.3
	Self	**-**	**-**	**-**	**1.3**	**-**	**0.3**	**-**	**-**
					0.1–10.9		0.04–1.9		
	Other	**-**	**-**	**-**	**23**	**-**	**-**	**-**	**4.5**
					2.2–80.1				2.5–8.1
Where did you get this medication?	Public system network	**75.5**	**87.8**	**90.5**	**63.6**	**96.9**	**66.4**	**74.6**	**6.6**
		57.3–87.6	56.6–97.6	38.0–99.3	23.8–90.7	96.9–96.9	54.8–76.4	60.4–85.0	4.9–9.0
	Pharmacy with ISAPRE card or clinic	**0.4**	**6.4**	**9.5**	**-**	**-**	**4.4**	**0.1**	**2.4**
		0.1–3.3	0.6–43.9	0.7–62			1.1–16.7	0.01–0.4	0.3–16,0
	Retail pharmacy	**21.7**	**5.00**	**-**	**36.4**	**3.1**	**25.4**	**24.4**	**83.4**
		10.2–40.4	0.7–30.0		9.3–76.2	3.1–3.1	16.5–37.0	14.0–39.0	63.1–93.7
	Other place (fair, street)	**-**	**-**	**-**	**-**	**-**	**0.7**	**-**	**0.3**
							0.1–4.8		0.04–2.6
	Gift from an acquaintance	**-**	**-**	**-**	**-**	**-**	**-**	**-**	**-**
	Given by the AUGE/GES	**2.3**	**0.7**	**-**	**-**	**-**	**1.9**	**0.8**	**7.2**
		0.4–13.3	0.4–1.5				0.4–8.6	0.1–5.7	1–38.2
	Other	**-**	**-**	**-**	**-**	**-**	**1.1**	**0.1**	**0.1**
							0.2–7.7	0.03–0.6	0.01–0.6
**FONASA**									
Who prescribed this medication?	General practitioner	**91.0**	**100**	**76.0**	**100**	**100**	**96.2**	**98.6**	**66.3**
		78.0–96.7	100–100	52.4–90.1	100–100	100–100	92–98.2	95.7–99.5	37.5–86.6
	Other specialist physician	**9.0**	**-**	**24.0**	**-**	**-**	**3.5**	**1.4**	**21.5**
		3.3–22		9.9–47.6			1.6–7.7	0.5–4.3	5.5–56.5
	Self	**-**	**-**	**-**	**-**	**-**	**0.3**	**-**	**-**
							0.0–2.3		
	Other	**-**	**-**	**-**	**-**	**-**	**-**	**-**	**12.2**
									6.6–21.3
Where did you get this medication?	Public system network	**91.1**	**94.6**	**90.5**	**83.6**	**99.7**	**76.2**	**77.0**	**3.6**
		81.4–96.0	67.8–99.3	38.0–99.3	78.0–87.9	99.7–99.7	63.3–85.6	61.5–87.5	2.0–6.4
	Pharmacy with ISAPRE card or clinic	**-**	**-**	**-**	**-**	**-**	**-**	**-**	**0.4**
									0.0–3.6
	Retail pharmacy	**6.2**	**5.4**	**9.5**	**16.4**	**0.3**	**21.1**	**22.2**	**95.8**
		2.9–12.6	0.7–32.2	0.7–62	12.1–22.0	0.3–0.3	12.0–34.3	11.7–38.1	92.4–97.7
	Other place (fair, street)	**-**	**-**	**-**	**-**	**-**	**0.8**	**-**	**-**
							0.1–5.7		
	Gift from an acquaintance	**-**	**-**	**-**	**-**	**-**	**-**	**-**	**-**
	Given by the GES	**2.8**	**-**	**-**	**-**	**-**	**0.5**	**0.9**	**-**
		0.4–15.8					0.1–2.1	0.1–6.1	
	Other	**-**	**-**	**-**	**-**	**-**	**1.3**	**-**	**0.3**
							0.2–9.2		0.03–2.3
**ISAPRE**									
Who prescribed this medication?	General practitioner	**10.2**	**10.5**	**-**	**-**	**-**	**52.1**	**59.7**	**43.6**
		1.9–39.9	1.2–53.0				17.4–84.9	12.1–94.1	12.2–81.2
	Other specialist physician	**89.8**	**89.5**	**-**	**26.7**	**-**	**47.9**	**40.3**	**53.9**
		60.1–98.1	47.0–98.8		0.6–95.6		15.1–82.6	5.9–87.9	17.4–86.7
	Self	**-**	**-**	**-**	**-**	**-**	**-**	**-**	**-**
	Other	**-**	**-**	**-**	**73.3**	**-**	**-**	**-**	**2.5**
					4.4–99.4				0.3–18.8
Where did you get this medication?	Public system network	**-**	**-**	**-**	**-**	**-**	**1.6**	**-**	**-**
							0.2–11.8		
	Pharmacy with ISAPRE card or doctor’s office	**2.7**	**89.5**	**-**	**-**	**-**	**35.6**	**5.5**	**4.9**
		0.3–20.7	47.0–98.8				7.4–79.3	0.6–37.0	0.6–32.5
	Retail pharmacy	**97.3**	**-**	**-**	**100**	**-**	**47.6**	**94.5**	**78.9**
		79.3–99.7			100–100		15.9–81.4	63.0–99.4	35.8–96.2
	Other place (fair, street)	**-**	**-**	**-**	**-**	**-**	**-**	**-**	**0.7**
									0.1–5.9
	Gift from an acquaintance	**-**	**-**	**-**	**-**	**-**	**-**	**-**	**-**
	Given by the GES	**-**	**10.5**	**-**	**-**	**-**	**15.2**	**-**	**15.5**
			1.2–53.0				2.0–61.2		1.8–64.6

Source: Prepared by authors, based on NHS database 2016/2017.

Among those suffering from dyslipidemia, the most-reported medications for the disease were Atorvastatin (C10AA05), used by 8.4%, and Gemfibrozil (C10AA07), used by 3.6%, both to lower cholesterol and/or triglyceride levels [27]. Atorvastatin registered 6.6% among FONASA beneficiaries and 33.1% in ISAPRE beneficiaries, while Gemfibrozil was indicated by 3.9% in FONASA beneficiaries (ISAPRE beneficiaries only indicated Atorvastatin).

Most of these medications were prescribed by a general practitioner, although in the case of ISAPRE members, the mention of the "other" category regarding the prescription of Atorvastatin is striking. Regarding its obtaining, Atorvastatin was acquired in the public network or in pharmacies in a private way; while the first modality was the most recurrent among the FONASA beneficiaries, the second was indicated in 100% of the ISAPRE cases; Gemfibrozil was obtained mostly in the public network (FONASA).

Finally, the medications for AHT most used by those with this condition were Losartan (C09CA01), angiotensin II receptor antagonist [27], with 23.7%, Enalapril (C09AA02), which is an ACE inhibitor [27], with 9.3%, Hydrochlorothiazide (C03AA03) which is a diuretic thiazide indicated for hypertension [27], with 3.6%, and Valsartan (C09CA03), which inhibits the effect of angiotensin II on blood pressure [27], with 3.5%.

In the case of Losartan, the usage figures were similar between FONASA members (24.1%), and ISAPRE ones (21%). Enalapril was consumed mainly by FONASA beneficiaries (10.5%) and by less than 1% of those who contributed to the private system (0.9%). Hydrochlorothiazide was used by 2.9% of FONASA members (1.7–4.8%) and by 9.7% of ISAPRE members. In the case of Valsartan, its use was more frequent in ISAPRE (15.4%) than in FONASA (1.2%).

Other medications indicated, but with a lower use were beta-blockers [27], called Atenolol (C07AB03), with 2.6% (95%CI 1.5–4.6%; FONASA 2.5%, 95%CI 1.3–4.7% and ISAPRE 0.1%, 95%CI 0–0.5%), and Carvedilol (C07AG02), with 1.8% (95%CI 0.7–4.7%; FONASA 0.7%, 95%CI 0.3–1.6% and ISAPRE 7.4%, 95%CI 1.1–37.3%); Nifedipine (C08CA05), which blocks calcium channels [27] with 1.6% (95%CI 0.9–2.9%; FONASA 1.9%, 95%CI 1–3.4% and ISAPRE 0.4%, 95%CI 0–2.7%) and Candesartan (C09CA06), angiotensin II receptor antagonist [27], mentioned by 4.7% of ISAPRE members (95%CI 1.1–18.18%).

Regarding their prescription, considering Losartan, Enalapril and Valsartan, the medications were mainly prescribed by a general practitioner, especially in the case of FONASA beneficiaries. In ISAPRE members, the participation of medical specialists reached up to 53.9% in the prescription of Valsartan; this medicine was bought in pharmacies by most of those who used it, regardless of whether they had FONASA or ISAPRE. Losartan and Enalapril were mainly acquired in the public network, a situation determined above all by the FONASA beneficiaries, since the ISAPRE members acquired them in pharmacies (Table 4).

The mention of GES was also very low in medicines for this disease, although it reached 15% in Losartan and Valsartan among ISAPRE members (Table 4).

## Discussion

The prevalence of the three conditions studied remains high in Chile and about 3.5% of people aged 15 years or older have all three conditions together. Although the prevalence of diabetes is the lowest, it has registered a sustained increase since 2003 [19], while hypertension decreased compared to 2003, but seems to have stabilized relative to the 2010 measurement [10, 20]. For its part, dyslipidemia has the highest prevalence (measured in terms of high total cholesterol), affecting almost a third of the population.

In contrast, the medication use is higher in the case of diabetes and very low among those with dyslipidemia, and in all diseases, at least 40% of those who suffer from them, do not use medications, which shows that, despite interventions such as GES [15] and FOFAR [16], medication use remains low. It is not surprising, then, that effective coverages are low as well. Although they exceed those recorded in 2009 [10, 19, 20], only in the case of diabetes, effective coverage is achieved in more than half of patients. Situation consistent with what was reported in terms of hypertension for Latin America by Lamelas et al [28]. Although reasons behind the drop from medication use to effective coverage in the case of hypertension and dyslipidemia were not explored in this research, adherence to medication might have some influence [29].

The relationship between medication use and effective coverage appears to be more evident in hypertension and dyslipidemia, showing significant differences between those who use medications and those who do not. However, in the case of diabetes, a trend is observed but does not reach statistical significance, possibly due to the significance of non-pharmacological treatments for disease control, as reported by 22% in the 2016–2017 National Health Survey [19].

Other factors that favor the use of medications in our analysis are female sex and being 65 years or older, already reported in previous studies on diabetes [19] and hypertension in Chile [10, 20]. In addition, in the case of this last condition, a greater use was found in people with 13 or more years of study compared to those with less than 8 years of education.

Only in the case of hypertension, women exhibit significantly higher effective coverage than men, which is consistent with the findings of Passi et al [10]. There are no clear patterns according to age and highlights the difference in favor of users of the private health system (ISAPRE), both in diabetes and hypertension. Those in the private system might have better healthcare management or better adherence, which, in turn, could be associated with social determinants of health [22]. However, this study does not allow for an in-depth exploration of the underlying mechanisms behind the differences found.

These differences are also recorded in the way drugs are accessed. The segmentation of the Chilean health system, in terms of institutionalism, processes, financing, insurance and provision, in addition to the structure of the pharmaceutical market, the characteristics of the local industry and the geographical distribution of health centers and pharmacies, translate into different ways of obtaining and using medicines [6], when determining the existence or not of availability barriers, financial, adaptation (e.g. through flexible dispensing schedules) and acceptability coverage, among others, which have been shown to hinder access [5].

Thus, while almost all the beneficiaries of the public system (FONASA) are prescribed medicines by a general practitioner and obtain them in the public network facilities; in those who have ISAPRE (private system), the prescription comes from specialist physicians and the purchase in retail pharmacies predominates with the consequent out-of-pocket expense. In addition, in both the public and private systems, strategies such as the GES have a low mention, reflecting low use or ignorance of the origin of the medicines provided.

Less differentiation is observed in the medicines most used by patients, which are consistent with the recommendations of current clinical guidelines [26, 30–32]: Metformin in diabetes, Atorvastatin in dyslipidemia and Losartan in hypertension. Only in some drugs, such as Enalapril and Valsartan, it is possible to note differences according to the health system; while the first is consumed mainly by FONASA members, the second is consumed by ISAPRE members.

This analysis has the strength of using the most recent official data of the country, however, the data of the same, which refer to 2016–2017, can be pointed out as a limitation. In addition, the results are not directly extrapolated to other countries, although segmentation is a common characteristic among Latin American health systems [33].

In this context, the challenge is to periodically update the indicators of medication use and effective coverage, based on systematic approaches that allow improving the detection and optimization of treatment [27], that are appropriate to the reality of each country and that allow monitoring the advances in the control of conditions of high prevalence and impact on health, such as those studied.

Finally, further research allowing in-depth interpretation of these results, such as the greater use of medications for some groups like women and elder, or the adherence to medications and its impact on the effective coverage are needed.

## Conclusions

Although the medications used are consistent with existing recommendations in the country and strategies have been developed to increase access to them, a significant proportion of people with hypertension, diabetes and dyslipidemia still do not use the required medicines and, moreover, do not control their conditions. It is necessary to periodically monitor coverage and comprehensively analyze barriers to access and use, to improve the control of these diseases.

## Supporting information

S1 File (DOCX)
