# Supplementary material for: Prescription drug coverage and effective coverage of three chronic conditions of high prevalence in Chile: Hypertension, diabetes and dyslipidemia
Source: PLoS One. 2024 Feb 12;19(2):e0297807. doi: 10.1371/journal.pone.0297807 (PMC10861056; doi:10.1371/journal.pone.0297807)
Supplement: S1 File — (DOCX) [file pone.0297807.s001.docx]

The two databases (Base Formulario 1-Formulario 2 y exámenes – comuna y variables complejas (SPSS) and Base de Medicamentos – Base 2 medicamentos) were downloaded from the following link: <http://epi.minsal.cl/bases-de-datos/>

To construct the prevalences of AHT, DM, and DLP, according to the criteria described in the article (Variables and Instruments section), the variables following variables (from Base Formulario 1-Formulario 2 y exámenes) were used **HTA** (for AHT) and **DMTayuno** (for DM), both straight from the database. In order to construct DLP prevalence, the variables used were: Colesterol_LDL_Calculado, **dis6==1** or **dis6==3** (pharmaceutical treatment for dyslipidemia), **RCV_CHILENO_RECODIFICADO==3** (high cardiovascular risk) and **d1_F1==1** (self-reported previous myocardial infarction).

Regarding Medication use, in the case of AHT the variables were **h6==1** or **h6==3**; in the case of DM **di7_1==1** or **di7_2==1**; and for DLP, variables were **dis6==1** or **dis6==3.**

For Effective coverage, according to what is described in the article, the variables considered were: **m2p11a_PAS>=140** or **m2p11a_PAD>=90** for AHT, **Glucosa<126** and **Horas_ayuno>=8** for DM, and **Colesterol_LDL_Calculado<70** for DLP.

In order to consider complex simples, the variables used were:

AHT: **Fexp_F1F2p_Corr** (weight)**, estrato** and **conglomerado**.

Dislipidemia: **Fexp_F1F2_EX2p_Corr**  (weight)**, estrato** and **conglomerado.**

DM: **Fexp_F1F2_EX1p_Corr** (weight), **estrato** and **conglomerado**.

The demographic and socioeconomic variables: **Sexo** (sex), **Edad** (age), **Zona** (residential area), **Est_civil** (having or not a partner), **c6** (belonging an indigenous people), **NEDU1_MINSAL_1** (educational level), **as5_1** (health system).

Finally, for the characterization of medication usage, the two databases were merged (using the variable **IdEncuesta** as ID). In this case, the variables used were: **ATC1** (ATC code)**, NOMBREPA1** (medication name), **m10E** (who prescribed), **m10F** (where was it obtained).
